# Transcriptional Signatures of Aerobic Exercise-Induced Muscle Adaptations in Humans

**DOI:** 10.3390/jfmk10030281

**Published:** 2025-07-19

**Authors:** Pranav Iyer, Diana M. Asante, Sagar Vyavahare, Lee Tae Jin, Pankaj Ahluwalia, Ravindra Kolhe, Hari Kashyap, Carlos Isales, Sadanand Fulzele

**Affiliations:** 1 Department of Medicine, Augusta University, Augusta, GA 30909, USA; piyer@augusta.edu (P.I.); cisales@augusta.edu (C.I.); 2 Department of Cell Biology and Anatomy, Augusta University, Augusta, GA 30909, USA; dasante@augusta.edu; 3 Division of Biostatistics and Data Science, Augusta University, Augusta, GA 30909, USA; 4 Department of Pathology, Augusta University, Augusta, GA 30909, USA; pahluwalia@augusta.edu (P.A.); rkolhe@augusta.edu (R.K.); 5 Department of Occupational Therapy, Augusta University, Augusta, GA 30909, USA; hkashyap@augusta.edu; 6 Center for Healthy Aging, Augusta University, Augusta, GA 30909, USA; 7 Department of Orthopaedic Surgery, Augusta University, Augusta, GA 30909, USA; 8 Department of Neuroscience & Regenerative Medicine, Medical College of Georgia, Augusta University, Augusta, GA 30912, USA

**Keywords:** muscle, exercise, transcriptional

## Abstract

**Background**: Aerobic exercise induces a range of complex molecular adaptations in skeletal muscle. However, a complete understanding of the specific transcriptional changes following exercise warrants further research. **Methods**: This study aimed to identify gene expression patterns following acute aerobic exercise by analyzing Gene Expression Omnibus (GEO) datasets. We performed a comparative analysis of transcriptional profiles of related genes in two independent studies, focusing on both established and novel genes involved in muscle physiology. **Results**: Our analysis revealed ten consistently upregulated and eight downregulated genes across both datasets. The upregulated genes were predominantly associated with mitochondrial function and cellular respiration, including MDH1, ATP5MC1, ATP5IB, and ATP5F1A. Conversely, downregulated genes such as YTHDC1, CDK5RAP2, and PALS2 were implicated in vascular structure and cellular organization. Importantly, our findings also revealed novel exercise-responsive genes not previously characterized in this context. Among these, MRPL41 and VEGF were significantly upregulated and are associated with p53-mediated apoptotic signaling and fatty acid metabolism, respectively. Novel downregulated genes included LIMCH1, CMYA5, and FOXJ3, which are putatively involved in cytoskeletal dynamics and muscle fiber type specification. **Conclusions**: These findings enhance our understanding of the transcriptional landscape of skeletal muscle following acute aerobic exercise and identify novel molecular targets for further investigation in the fields of exercise physiology and metabolic health.

## 1. Introduction

Aerobic exercise, or endurance exercise, is a physical activity that increases our heart rate over an extended period of time [1]. In addition to its effects on aerobic capacity, endurance exercise has proven to be an effective method in improving cardiovascular risk factors, glucose tolerance, muscle strength, mood, and overall longevity [2]. At a molecular level, exercise induces adaptations in various signaling pathways and processes such as increased mitochondrial biogenesis, improved oxidative capacity, and enhanced lipid metabolism [3]. Gradually, these adaptations accumulate, leading to an overall improvement in health and a decreased risk of chronic diseases such as cardiovascular disease, hypertension, and diabetes.

Recent studies have demonstrated that exercise alters the transcriptional profiles that positively affect cell remodeling, mitochondrial density, growth factors, and immune response modulation. For example, following eccentric exercise, Hyldahl, Xin [4] demonstrated activation of the Nf-Kb transcription factor in human muscle, highlighting its role in the regeneration and inflammatory response. In the context of cancer, exercise has shown to be a highly effective therapy. Kurz, Hirsch [5] analyzed the tumor microenvironment in mice following aerobic exercise and found that it promoted the mobilization and accumulation of interleukin-15 (IL-15) and enhanced the infiltrative capacity of CD8+ T cells. These changes were associated with an improved anti-tumor immune response, demonstrating the potential of exercise to improve cancer immunotherapy [5]. Exercise has also been shown to drive positive changes in neurological functions. Alvarez-Saavedra, De Repentigny [6] reported that voluntary running in mice exhibited increased VGF, which promoted an increase in oligodendrogenesis and modulated the serotonin, dopamine, and norepinephrine expression. This suggests that exercise can improve mood and motor function [6].

Identifying the specific molecular mechanisms and physiological adaptations that occur following exercise remains a key area of investigation. While numerous studies have identified alterations in gene expression, protein synthesis, and signaling pathways, the exact mechanisms underlying these changes remain largely unknown [7]. One of the challenges is the tendency of investigators to focus on highly significant genes or their specific genes of interest. This approach can lead to an incomplete picture of the molecular landscape because it often overlooks the subtle yet consistent changes in the transcriptome that occur in response to exercise. These changes could be involved in fine-tuning cellular processes such as energy metabolism, mitochondrial biogenesis, and muscle fiber composition, which are critical for enhancing endurance capacity. Ignoring these small changes may result in missing important insights into how the body responds to and benefits from endurance training. In individual studies, such microscopic adaptations could be biologically meaningful. A more holistic approach is needed to understand exercise-induced molecular changes. This might involve integrating data from multiple studies to identify common genes that responded to exercise. Considering the common major and minor changes in the transcriptome of various studies can help develop a more comprehensive understanding of how endurance exercise shapes skeletal muscle function and overall health.

In this study, we aim to advance the field by conducting a comprehensive analysis of existing data to identify consistent patterns in gene expression following exercise. We analyze two independent studies investigating the effects of aerobic and high-intensity exercise (GSE221894, GSE151066) on skeletal muscles. In doing so, we identified several known and unknown genes that play important roles in muscle physiology.

## 2. Materials and Methods

We utilized the Gene Expression Omnibus (GEO) database to identify studies examining the impact of exercise on gene regulation. Our search was refined to focus specifically on acute bouts of aerobic exercise and their effects on transcriptional profiles. Using keywords of “aerobic exercise,” “mRNA,” and “transcription,” we specifically searched for studies researching the effects aerobic exercise in both males and female humans who were studied before and after exercise training programs. Further, we focused on studies that performed total RNA isolation via skeletal muscle biopsies before and after interventions to determine changes in transcription following exercise. Three studies (GSE59088, GSE151066, GSE221894) meeting these criteria were selected for further analysis. One study (GSE59088) did not have sequencing data from the muscle biopsies, so only two studies (GSE151066, GSE221894) were analyzed to assess the gene expression profiles following exercise intervention. GSE221894 examined 15 male and female participants aged 18–45 years old with a body mass index between 18 and 25 kg/m^2^ and sought to determine changes in mitochondrial remodeling via skeletal muscle biopsy samples before and after 7 sessions of high-intensity interval training (HIIT) over 14 days. For each training session, participants completed 10 intervals of cycling for 1 min (~90% VO2 max) followed by 1 min of rest. GSE151066 recruited older men and women 65–90 years of age with no chronic medical conditions or contraindications for exercise and assigned the participants to an active/endurance trained group (n = 10; 8 males, 2 females) or a sedentary group (n = 9; 7 males, 2 females). Here participants in the active/endurance trained group were assigned to an aerobic endurance program (running, cycling and/or swimming) for ≥3 days a week, while the participants in the sedentary group engaged in exercise ≤ 1 day a week. Along with self-reported exercise habits, objective exercise data was obtained via vastus lateralis biopsy specimens during one of the visits. During this visit, biopsies were obtained initially as a baseline, immediately post-exercise, and 3 h following the exercise bout (40 min cycling exercise at 60–70% heart rate). RNA sequencing was performed on the biopsies in both studies to determine changes in transcription. We then systematically identified upregulated and downregulated genes across these studies and conducted a comparative analysis of their transcriptional profiles (Table 1).

**Figure 1:**
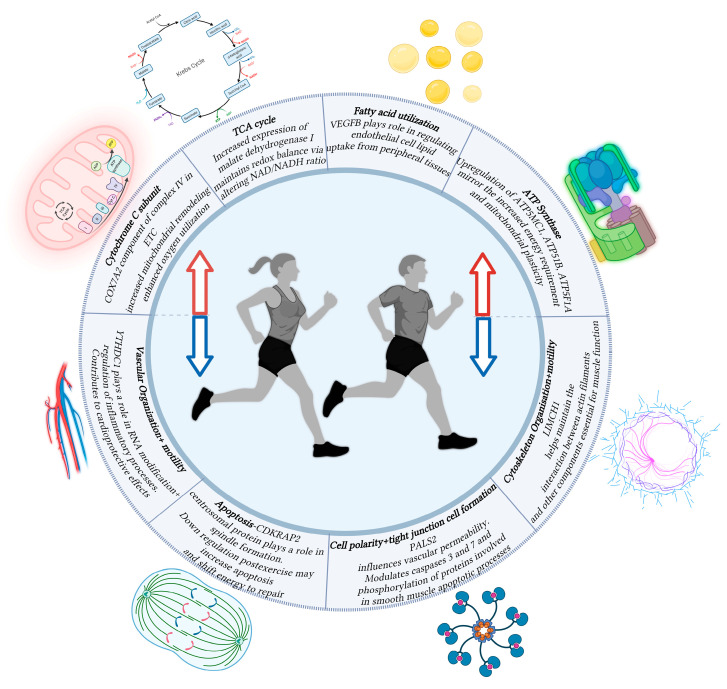
Schematic diagram showing the function of various genes associated with changes in transcription following aerobic exercise. Increased transcription (Red Arrow) rates were observed in genes relating to the TCA cycle, fatty acid utilization, and processes relating to oxidative phosphorylation. Decreased transcription (Blue Arrow) rates were observed in genes relating to vascular organization, cell apoptosis, cellular polarity, and cellular motility. Created in BioRender. Asante, D. (2025) https://BioRender.com/jymv8rl.

Data Selection and Statistical Analysis: We analyzed publicly available transcriptomic datasets obtained from the Gene Expression Omnibus (GEO), specifically focusing on GSE151066 and GSE221894. For each study, we downloaded the processed differential expression results, including log fold change (logFC) values and adjusted *p*-values, as provided by the original studies. To identify genes of interest, we applied the following criteria: an adjusted *p*-value < 0.05 to ensure statistical significance, and opposing logFC directions between the two studies, indicating a potential divergence in transcriptional response based on age, training status, or other study-specific factors. Comparative analysis of the transcriptional profiles across the two studies revealed commonalities in both upregulated and downregulated genes. Furthermore, we identified a subset of genes that were consistently modulated post-exercise across all studies but were not previously reported. These novel commonalities are addressed and discussed in detail herein. We also performed pathway analysis, illustrated in Figure 1.

## 3. Results and Discussion

The analysis of transcriptional profiles between the two studies revealed 10 consistently upregulated genes and 8 downregulated genes across the studies. Notably, we found that many of these genes are novel, with no prior reports linking them to exercise. The reported genes were found to be involved in a variety of signaling pathways, including mitochondrial biogenesis, cellular respiration, cytoskeletal organization, and vascular reorganization (Figure 1). The involved genes are discussed in detail below along with their potential implications of their changes in regulation post-exercise.

### 3.1. Elevated Gene Expression Involved in Cellular Respiration Following Aerobic Exercise

The transcriptional profiles of several genes involved in cellular respiration were elevated following aerobic exercise. Malate dehydrogenase 1 (MDH1), a key enzyme in the tricarboxylic acid cycle [8], showed an increase in expression, likely due to the heightened energy demand and oxidative stress response during exercise. These elevated levels may contribute to maintaining redox balance by altering the NAD/NADH ratio. Multiple genes encoding mitochondrial ATP synthase subunits were upregulated, including ATP5MC1, ATP51B, and ATP5F1A. ATP5MC1 and ATP51B encode components of the proton channel and the soluble catalytic core, respectively [9], while ATP5F1A encodes the alpha subunit of the F1 catalytic core [10]. These changes reflect the increased energy requirements and mitochondrial plasticity observed following exercise. Additionally, cytochrome c oxidase subunit 7A2, a member of complex IV in the electron transport chain, showed an increase in expression. This upregulation is consistent with the observed increase in mitochondrial remodeling and enhanced oxygen utilization seen in prolonged aerobic exercise. It is likely that aerobic exercise mediates this change in gene expression through activation of regulators such as AMPK and PGC1-α, which promote mitochondrial biogenesis [11] and therefore an increased capacity to produce energy. We also found that coenzyme Q10 was elevated following exercise, which plays a role in oxidative phosphorylation [12] and reduces oxidative stress associated with fatigue and inflammation.

### 3.2. Aerobic Exercise Alter Genes Involved in Vascular and Cellular Organization

Several genes involved in vascular and cellular organization were found to be altered following aerobic exercise. YTHDC1, which plays a role in RNA modification and regulation of inflammatory processes, was downregulated in both studies. Specifically, YTHDC1 is involved in m6A RNA methylation [13], which can influence gene expression and inflammatory pathways. Recently, Yang, Chen [14] demonstrated that aerobic exercise led to a downregulation of the long-coding RNA NEAT1 via decreased expression of the YTHDC1 domain in a mice model. This suggests a potential reduction in atherosclerosis-related inflammatory processes post-exercise due to the involvement of RNA NEAT1 in the secretion of pro-inflammatory markers IL-1B and IL-18 [14]. These findings imply that aerobic exercise may contribute to cardioprotective effects by modulating YTHDC1 and attenuating inflammatory processes linked to vascular pathology.

Glycerol-3-phosphate dehydrogenase 2 (GPD2) showed a decreased expression in both GeoData sets, which may modulate the inflammatory response and alter metabolic pathways, favoring more efficient ATP production during prolonged exercise. GPD2 encodes an enzyme that is involved in glycerol metabolism and plays a role in the mitochondrial glycerophosphate shuttle, which links glycolysis to oxidative phosphorylation [15]. It has been previously reported that GPD2 indirectly plays a role in the inflammatory response by fueling the production of acetyl CoA. This increased production can provide substrates for histone acetylation at various promoters of inflammatory mediators [16]. While this mechanism was only outlined in immune cells activated by bacterial lipopolysaccharides, we propose that GPD2 could similarly regulate the immune response by contributing to the observed anti-inflammatory effects following exercise. Further, its downregulation might shift cellular metabolism away from traditional pathways and towards more efficient energy production mechanisms, such as increased reliance on fatty acid oxidation, which is more sustainable during prolonged exercise.

### 3.3. Regulation of Apoptosis Signaling Pathways Post-Exercise

Apoptosis is a highly regulated form of programmed cell death critical for the maintenance of cellular homeostasis and tissue integrity [17]. This process is characterized by several distinct morphological and biochemical changes, which include shrinkage of the cell and its nucleus, chromatin condensation, and DNA fragmentation [18]. Under normal physiological conditions, apoptosis eliminates damaged, senescent, or otherwise potentially injurious cells, which prevent the accumulation of dysfunctional cellular components and reduce the risk of dysplastic proliferation. In the context of exercise, however, the role of apoptosis remains an area of interest and ongoing investigation.

In our data analysis, we identified two genes (MRPL41, CDK5RAP2) associated with apoptosis pathways altered with aerobic exercise. MRPL41 is involved in the apoptosis-p53 signaling pathway, which is crucial for cellular stress responses [19]. Our analysis showed upregulation of MRPL41 in both GeoData sets. The observed increase in MRPL41 could be due to several reasons, one being to meet the increased energy demand through increasing protein synthesis and cellular respiration. Exercise can induce a transient increase in oxidative stress which triggers cellular defense mechanisms, including the activation of p53. In response to the increased oxidative stress, it is possible that MRPL41 and its subsequent involvement in the p53 pathway could be upregulated to promote cellular repair and adaptation. In addition, aerobic exercise often creates a hypoxic environment within skeletal muscle due to its high oxygen demand [20]. Hypoxic environments have been shown to repress myogenic differentiation by upregulating p53-dependent pathways [19]. It is possible that the hypoxic environment created following aerobic exercise could influence an upregulation in the MLRP41 gene to activate p53-related pathways to reduce myoblast differentiation temporarily. Reducing differentiation could be due to energy allocation (shifting energy towards immediate recovery rather than myoblast differentiation), a response to acute stress caused by exercise, or a response to a metabolic shift (post-exercise states may favor carbohydrate and fat utilization rather than anabolic processes like myoblast differentiation).

MRPL41 also plays a critical role in mitochondrial protein synthesis, which is essential for maintaining mitochondrial function and energy production. MRPL41 encodes a mitochondrial ribosomal protein part of the mitochondrial ribosome’s large subunit [21]. Aerobic exercise stimulates mitochondrial biogenesis, the process by which new mitochondria are formed in the cells. The demand for increased ATP production during exercise leads to enhanced mitochondrial function and efficiency. The regulation of MRPL41 during exercise might reflect a shift in cellular metabolism to enhance energy efficiency and reduce oxidative damage which will support long-term mitochondrial health and improved endurance capacity.

Our data analysis showed a decrease in the expression of CDKRAP2 following the exercise. CDKRAP2 is a centrosomal protein that plays a role in centrosome cohesion and proper spindle formation during cell division [22]. It is also involved in DNA damage response and repair, helping cells maintain genomic stability [22]. While CDKRAP2 is most abundant in the brain, it is also expressed in other tissues and associated with developmental disorders such as primordial dwarfism and Seckel syndrome [23,24]. Recent studies regarding CDK5RAP2 have demonstrated its potential as a regulator of apoptosis through the regulation of CDK5 activity via its interaction with CDK5R1 [25]. Additionally, loss of CDK5RAP2 has been shown to cause lagging chromosomes and a disruption in overall chromosomal integrity through its interactions with CENP-A [26]. Its role in regulation following exercise or in skeletal muscle has not been studied, but its mechanisms involving structural and chromosomal integrity point to its potential involvement in apoptotic mechanisms observed following exercise. Exercise, particularly intense or prolonged aerobic activity, is known to induce a certain degree of cellular stress, DNA damage, and apoptosis in skeletal muscle as part of the adaptive response [27]. CDK5RAP2’s role in proper spindle formation suggests that its expression might be downregulated to promote cell cycle arrest and shift its resources toward healing and regeneration rather than cell division. This could prevent the proliferation of damaged muscle cells and ensure that cell division is tightly regulated. By temporarily halting cell division, the body can also focus on repairing and adapting to the physical stress, leading to improved tissue resilience and function. Another potential explanation for the downregulation of CDK5RAP2 could be related to its role in maintaining chromosomal integrity. Studies have shown that loss of CDK5RAP2 can lead to lagging chromosomes and disruption of overall chromosomal integrity through its interactions with centromere protein A, also known as CENPA [26]. In the context of exercise, where cellular stress and DNA damage can occur, the downregulation of CDK5RAP2 might be part of a coordinated response to halt division and allow for the body to respond to the physical damage and oxidative stress produced during exercise. These functions of CDK5RAP2 altogether might allow cellular resources to be redirected toward repair, recovery, and adaptation processes rather than cell division in the immediate post-exercise period. The decreased expression of CDK5RAP2 could therefore be a protective mechanism that modulates apoptosis during the recovery phase following exercise.

We also observed the PALS2 gene downregulated across two studies following the exercise. PALS2 (Protein Associated with Lin Seven 2) is primarily known for its role in maintaining cell polarity and tight junction formation but it can also influence apoptosis, the process of programmed cell death [28]. Through genome-wide association studies (GWASs), McVey, Andreadi [28] found PALS influences the regulation of cleaved/activated caspase 3 and 7 levels and the phosphorylation of various proteins involved in the vascular smooth muscle (SM) apoptotic processes. Downregulation of PALS2 might reduce the integrity of tight junctions, leading to increased vascular permeability. This can be beneficial during exercise as it allows for enhanced delivery of oxygen, nutrients, and metabolic substrates to the muscles, which are in high demand during physical activity. However, further studies are needed to elucidate its role following exercise.

### 3.4. Increased Fatty Acid Utilization Post-Exercise Through VEGFB Regulation

GeoData set analysis showed elevated Vascular endothelial growth factor B (VEGFB) levels. Vascular endothelial growth factor B (located on chromosome 11q13.1) is one of six growth factors in the VEGF family, which play crucial roles in vascular biology and metabolism [29]. VEGFB has previously been shown to have a role in endothelial angiogenesis in mouse modules through interaction with its receptor VEGFR1 and downstream pathways of AKT and eNOS [30]. VEGB has also been shown to have a role in the endothelial uptake of fatty acids metabolized by tissue cells.

Specifically, Hagberg, Falkevall [31] showed that VEGF-B knockout mice showed less accumulation of lipids in muscle, heart, and brown tissue and evidence of increased shunting to white adipose fat. Hagberg, Falkevall [31] found that VEGF-B plays a substantial role in regulating endothelial lipid uptake from peripheral tissues such as skeletal muscle, cardiac muscle, and brown adipose tissue. They also showed an increased expression of GLUT4 in VEGFB-knocked-out mice. Here we found VEGF-B to be upregulated following aerobic exercise. Following exercise, our body has less of a need for increased glucose uptake, so VEGFB could be upregulated to lower the expression of GLUT4 to the heart and skeletal muscle cells. Additionally, following exercise we can see a shift towards increased fatty acid uptake in peripheral tissues indicating a shift from the need for immediate energy to an anabolic state. This is based on the findings of Savage, Petersen [32] which demonstrate that reduced lipid accumulation in myocytes can enhance glucose use and insulin sensitivity due to decreased competition between fatty acids and glucose for substrates [32,33].

Given this current understanding of VEGFB’s functions, it is reasonable to suggest that this gene might be upregulated following aerobic exercise. The role of VEGF-B in endothelial cell growth and angiogenesis, mediated through VEGFR1 and neuropilin-1 [34], could support the increased vascularization that is typically observed with regular aerobic training. More intriguingly, VEGFB’s function in regulating lipid transport and distribution across peripheral tissues is particularly relevant to exercise metabolism. Following exercise, VEGF-B could facilitate the uptake and utilization of fatty acids in metabolically active tissues such as skeletal muscle and heart. This hypothesis is further supported by VEGFB’s observed co-expression with mitochondrial genes, suggesting it may play a role in the coordinated response to increase mitochondrial function a key adaptation to aerobic exercise. While these connections are compelling, direct measurement of VEGFB expression and activity in response to acute and chronic aerobic exercise is necessary to confirm this hypothesis and elucidate how VEGFB might contribute to exercise-induced metabolic and vascular adaptations.

### 3.5. Exercise Modulates Novel Genes (LIMCH1, CMYA5, FOXJ3) Involved in Muscle Physiology

Our analysis revealed several novel genes (LIMCH1, CMYA5, FOXJ3) that exhibited changes in transcription following aerobic exercise. FOXJ3 (Forkhead Box J3) is a transcription factor belonging to the Forkhead family of proteins, which regulate various biological processes involved in cell development, metabolism, and differentiation [35]. While studies on FOXJ3 are not as extensive compared to other FOX proteins, its role as a transcription factor suggests that its downregulation during exercise could have significant implications for muscle function and overall exercise adaptation. Alexander, Shi [36] investigated the expression of Foxj3 in adult skeletal muscle and demonstrated that the knockdown of FOXJ3 decreases Type I slow-twitch myofibers and impairs skeletal muscle contractile function. The study further showed that FOXJ3 was involved in regulating muscle fiber identity and regeneration of muscles in mice models [36]. Downregulation of FOXJ3 in our study following exercise could be associated with a shift in muscle fiber composition to promote a healthy balance between slow-twitch (oxidative) and fast-twitch (glycolytic) fibers that is most conducive to muscle healing and regeneration. This decrease in FOXJ3 would likely also lead to a reduction in MEF2C expression, a transcription factor involved in muscle development, given FOXJ3’s role as a direct activator of MEF2C [36]. While MEF2C is vital for maintaining Type I fibers, its downregulation might facilitate fiber type switching or adaptations beneficial for aerobic performance, like the direct actions of FOXJ3. Overall, this could improve muscle efficiency and endurance in future bouts of exercise. Since FOXJ3 also acts as an antiproliferative factor in muscle progenitor cells [36], its downregulation may promote increased proliferation and muscle regeneration in response to stress and damage introduced by aerobic exercise. Increased cell proliferation and decreased contractile function following exercise indicate a state of healing and regeneration of the muscle cells following aerobic exercise, and our analysis suggests that FOXJ3 might have a pivotal role in facilitating these processes.

Interestingly, we also found a downregulation in two genes, LIMCH1 and CYMA5, that are involved in cellular organization and motility. Kadrmas and Beckerle [37] demonstrated that LIMCH1 was specifically involved in cytoskeletal organization and motility. Here we found that LIMCH1 was downregulated following exercise, which could be part of the body’s recovery response to conserve energy and focus on regenerative processes. The actin cytoskeleton is crucial for muscle contraction, cell movement, and maintaining cell shape. LIMCH1 helps modulate the interaction between actin filaments and other cytoskeletal components, which is essential for muscle function [38]. During exercise, the cytoskeleton actively maintains muscle integrity under mechanical stress [39]. After exercise, the immediate demand for this structural support decreases, which could explain the downregulation of LIMCH1. This downregulation might help reduce energy expenditure associated with maintaining and remodeling the cytoskeleton, allowing energy to be redirected toward other recovery processes. Through this process, cells might prioritize repair and adaptation processes, ensuring that muscle and vascular tissues recover effectively from the physical stress imposed by exercise.

CMYA5 (Cardiomyopathy Associated 5), also known as myospryn, is associated with protein kinase A signaling and plays a role in muscle organization and function [40]. Previously, Sarparanta, Blandin [41] reported that CMYA5 interacts with titin and calpain 3, and is therefore involved in muscle contraction and structural regulation. Kielbasa, Reynolds [42] identified CMYA5 as a novel calcineurin-interacting protein that inhibits calcineurin-dependent transcriptional activation in vitro and calcineurin-mediated muscle remodeling in vivo. Negative regulation of calcineurin via CMYA5 is specifically associated with the slow-fiber gene expression program, a pathway typically activated by calcineurin [42]. Based on the above literature, CMYA5 is integral to muscle adaptation and regeneration and a decreased expression with exercise might be beneficial for maintaining muscle function and organization. However, the exact mechanisms of CMYA5 in muscle remodeling and response to exercise are still unknown and more research needs to be conducted. In conjunction with LIMCH1, its downregulation may indicate a coordinated shift in cellular dynamics post-exercise, which could involve cellular reorganization or regeneration. The exact role of the observed downregulation of CMYA5 and LIMCH1 post-exercise warrants further research however to determine its significance for future therapeutic contexts.

**Figure 2:**
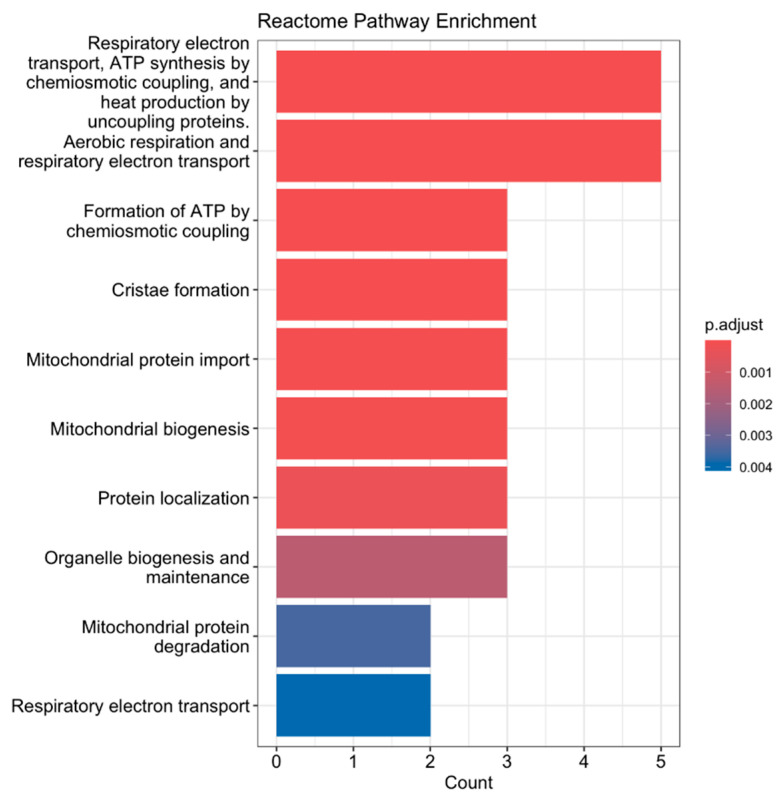
Reactome pathway enrichment analysis of differentially expressed genes following acute aerobic exercise. Genes significantly upregulated or downregulated in skeletal muscle post-exercise were analyzed using Reactome pathway enrichment analysis. The top enriched pathways are shown, ranked by log_10_(FDR-adjusted *p*-value). Key pathways include Respiratory Electron Transport, Aerobic Respiration, Mitochondrial Protein Import, and Protein Localization, indicating enhanced mitochondrial activity and energy metabolism.

We also performed a reactome pathway enrichment analysis (Figure 2), a bioinformatics method used to identify biological pathways that are statistically overrepresented (enriched) among a set of genes. Our analysis revealed that genes differentially expressed in response to exercise were significantly enriched in pathways related to respiratory electron transport, aerobic respiration, mitochondrial protein import, and protein localization, which was expected. These findings suggest that acute aerobic exercise induces transcriptional changes that enhance mitochondrial function and bioenergetic capacity. Specifically, the upregulation of genes involved in electron transport and oxidative phosphorylation reflects increased mitochondrial activity required to meet the elevated energy demands during exercise. Similarly, enrichment in mitochondrial protein import and localization pathways indicates enhanced mitochondrial biogenesis and remodeling, essential for maintaining efficient cellular respiration and energy production in skeletal muscle.

While our data analysis approach provides valuable insights into the transcriptional landscape following aerobic exercise, we acknowledge several important limitations. First, our analysis is purely conceptional and lacks the experimental validation needed to determine the significance of the observed changes in transcription. The inferences made based off our sequencing analysis reflect potential molecular mechanisms and should therefore not be used to infer definitive causal relationships. Rather, this study was aimed to shine light on potential novel mechanisms following exercise so further studies may build on these findings to determine true significance. Second, our study relies on existing datasets with different groups of participants and exercise protocols, which may introduce variability in our results. More studies should be conducted in controlled exercise setting with a similar population to assess our observed changes in transcription. Third, we cannot account for potential confounding variables such as nutritional factors, individual genetic variability, or environmental conditions that may have influenced each study. Taken together, these limitations highlight the need for carefully controlled, prospective studies to validate and extend our findings to make them clinically relevant.

An important constraint in our study design is the heterogeneity in exercise protocols between the two datasets analyzed. While the primary objective of this study was to explore transcriptional adaptations to aerobic exercise, we acknowledge that GSE221894 involved high-intensity interval training (HIIT) consisting of 10 one-minute cycling intervals at high intensity (~90% VO_2_max) rather than traditional steady-state aerobic exercise. This protocol predominantly utilizes anerobic metabolic pathways, which differs from the continuous moderate-intensity aerobic exercise typically associated with oxidative adaptations. Therefore, our findings cannot be attributed exclusively to aerobic exercise adaptations. However, the identification of consistently upregulated genes across both exercise modalities suggests that the observed changes could potentially reflect common transcriptional responses that occur regardless of exercise intensity or oxygen utilization. Notably, the upregulation of genes involved in mitochondrial function and cellular respiration (MDH1, ATP5MC1, ATP51B, and ATP5F1A. ATP5MC1, ATP51B, and COX7A2) is consistent with adaptations reported in both aerobic and high-intensity exercises, suggesting that these genes may be implicated in both respiration processes. Nevertheless, future studies should examine both aerobic and anaerobic exercise separately to better elucidate changes in transcription unique to each type of metabolism and validate our findings in a narrower exercise context.

It is important to note that increased mRNA expression does not always correlate with increased protein abundance. As extensively reviewed by [43], the relationship between mRNA and protein levels is often complex and non-linear. Following transcription, variations observed in protein levels can be accounted for by post-transcriptional regulations (protein and 3’ untranslated region lengths) along with the body’s ability to degrade/increase levels of the protein based on its current need [43]. Therefore, our findings should be interpreted as transcriptional responses rather than a definite correlation to protein levels following aerobic exercise. Further studies should assess the protein abundance following post-transcriptional changes to determine the true significance of our observed changes in transcription.

Nevertheless, our study can provide valuable insight to adaptations of our body in several significant ways. Through identifying consistent changes in transcription across different population and exercise modalities, our findings can provide a foundation for future studies to develop personalized treatment regimens to optimize exercise performance and recovery. The novel genes discussed (LIMCH1, FOXJ3, CMYA5) provide new avenues for developing potential biomarkers that could influence how our body promotes health benefits following exercise. Along with its anticipated benefit for exercise, the differences in transcription discussed can also provide insight into our body’s ability to adapt to various stressors introduced in the human body. Such changes could serve as valuable indicators when discussing treatments regarding chronic disease, aging, and other pathological processes.

## 4. Conclusions

Our GeoData set analysis of GSE151066 and GSE221894 revealed several consistent patterns of transcriptional regulation in response to exercise. We observed an elevation in expression of genes involved in cellular respiration, including MDH1, ATP5MC1, ATP51B, ATP5F1A, and cytochrome c oxidase subunit 7A2, reflecting the increased energy demands and mitochondrial plasticity associated with exercise. Conversely, we found downregulation of genes related to vascular and cellular organization, such as YTHDC1, CDKRAP2, PALS2, GPD2, and LIMCH1A, suggesting complex adaptations in vascular function and cellular remodeling. Notably, we identified novel changes in the expression of genes involved in apoptosis (MRPL41, CDK5RAP2) and fatty acid metabolism (VEGFB), which shed new light on the molecular mechanisms involved in adaptations following exercise. Collectively, these findings enhance our understanding of the physiological adaptations elicited by aerobic exercise and may provide a foundation for future investigations aimed at developing targeted therapeutic strategies for age-related chronic diseases. Nonetheless, additional studies are warranted to elucidate the functional relevance of these transcriptional changes in the context of systemic health and physical performance.

## Figures and Tables

**Table 1:** List of genes that were found to be consistently upregulated (blue) or down regulated (red) following aerobic exercise in two studies (GSE151066, GSE221894). A fold change greater than one indicates that transcription increased following aerobic exercise. Conversely, a fold change less than one indicates that transcription of the gene decreased following aerobic exercise.

Gene (Symbol)	GSE151066 FoldChange	GSE151066 padj	GSE221894 FoldChange	GSE221894 padj
MRPL41	2.409945	1.55E-35	1.518872	0.04875
GATD3	2.03637	1.52E-27	1.314032	0.03834
LOC102724023	2.022304	5.44E-32	1.312211	0.03763
MDH1	1.952064	2.66E-26	1.443929	0.00106
ATP5MC1	1.785094	3.31E-20	1.480413	0.02837
ATP5F1B	1.745935	1.59E-24	1.316767	0.03834
VEGFB	1.723092	1.80E-31	1.412254	0.02354
COX7A2	1.64376	7.12E-23	1.509426	0.04875
COQ10A	1.640346	5.11E-28	1.482467	0.00106
ATP5F1A	1.636938	1.43E-20	1.265757	0.0299
YTHDC1	0.772175	1.47E-24	0.831046	0.04875
CDK5RAP2	0.758384	9.56E-20	0.744323	0.04875
FOXJ3	0.756808	1.19E-18	0.833931	0.04875
SGCB	0.657016	1.60E-20	0.699308	0.04875
CMYA5	0.635957	3.98E-19	0.78404	0.04875
LIMCH1	0.594604	2.26E-37	0.789494	0.04875
GPD2	0.559419	1.97E-20	0.694959	0.04875
PALS2	0.495858	6.11E-19	0.619854	0.04002

## Data Availability

All data generated or analyzed during this study are included in the manuscript.
